# CYP1B1 inhibits ferroptosis and induces anti-PD-1 resistance by degrading ACSL4 in colorectal cancer

**DOI:** 10.1038/s41419-023-05803-2

**Published:** 2023-04-14

**Authors:** Congcong Chen, Yabing Yang, Yanguan Guo, Jiashuai He, Zuyang Chen, Shenghui Qiu, Yiran Zhang, Hui Ding, Jinghua Pan, Yunlong Pan

**Affiliations:** 1 Department of General Surgery, The First Affiliated Hospital of Jinan University, Guangzhou, China; 2 MOE Key Laboratory of Tumor Molecular Biology and Key Laboratory of Functional Protein Research of Guangdong Higher Education Institutes. Institute of Life and Health Engineering, Jinan University, Guangzhou, China

**Keywords:** Cancer immunotherapy, Prognostic markers

## Abstract

Immune checkpoint blockade (ICB) is a promising treatment strategy for colorectal cancer (CRC) patients. However, most CRC patients do not response well to ICB therapy. Increasing evidence indicates that ferroptosis plays a critical role in immunotherapy. ICB efficacy may be enhanced by inducing tumor ferroptosis. Cytochrome P450 1B1 (CYP1B1) is a metabolic enzyme that participates in arachidonic acid metabolism. However, the role of CYP1B1 in ferroptosis remains unclear. In this study, we demonstrated that CYP1B1 derived 20-HETE activated the protein kinase C pathway to increase FBXO10 expression, which in turn promoted the ubiquitination and degradation of acyl-CoA synthetase long-chain family member 4 (ACSL4), ultimately inducing tumor cells resistance to ferroptosis. Furthermore, inhibiting CYP1B1 sensitized tumor cells to anti-PD-1 antibody in a mouce model. In addition, CYP1B1 expression was negatively correlated with ACSL4 expression, and high expression indicates poor prognosis in CRC. Taken together, our work identified CYP1B1 as a potential biomarker for enhancing anti-PD-1 therapy in CRC.

## Introduction

Colorectal cancer (CRC) is one of the most common malignant tumors in the digestive system [1]. Based on the World Health Organization (WHO) mortality database from 1989–2016, the mortality rates as a result of colon and rectal cancer were predicted to increase to 60% and 71.5%, respectively, by 2035 [2]. At present, the treatment for CRC includes surgery, chemotherapy and targeted therapies. Recently, immune checkpoint blockade (ICB) has emerged as a promising therapy strategy for CRC patients. However, a small proportion of CRC patients benefit from ICB therapy. Hence, identifying more targets that can enhance the efficiency of ICB therapy is an important strategy for CRC treatment.

ICB promotes CD8^+^ T cell-mediated killing of tumor cells by blocking the interaction between PD-L1 and PD-1. Recently, CD8^+^ T cells reportedly induced ferroptosis in tumor cells via IFNγ in vivo [3–5]. Ferroptosis is a type of programmed cell death implicated in cancer therapy [6]. More importantly, inducing ferroptosis in tumor cells is an effective strategy to enhance anti-tumor immunotherapy [4, 7]. Therefore, this method presents a promising strategy to improve ICB efficacy by inducing tumor cell ferroptosis.

Cytochrome P450 1B1 (CYP1B1) is a heme-thiolate monooxygenase that is involved in metabolism, inflammation, angiogenesis and anticancer drug resistance [8, 9]. Interestingly, CYP1B1 is overexpressed in multiple malignant tumors [8, 10, 11]. However, it is unclear whether CYP1B1 is involved in ferroptosis or related to ICB therapy efficacy.

In this study, we proved that CYP1B1 promotes CRC cells resistance to ferroptosis, indicating that anti-PD-1 treatment efficacy may be enhanced by inhibiting CYP1B1. Mechanistically, CYP1B1 metabolizes arachidonic acid (AA) to 20- hydroxyeicosatetraenoic acid (20-HETE), which activates the protein kinase C (PKC) signaling pathway and promotes FBXO10 expression to increase poly-ubiquitination of ACSL4, thereby promoting ACSL4 degradation. CYP1B1 is highly expressed in CRC tumor tissues and is related to poor prognosis. Therefore, we believe that CYP1B1 may serve as a novel therapeutic target for CRC patients and may be useful for improving anti-PD-1 efficacy in CRC.

## Materials and methods

### Reagents and antibodies

Anti-β-Actin (sc-47778), anti-HA (sc-57592) antibodies were purchased from Santa Cruz biotechnology (TX, USA); anti-Flag (F1804) antibodies were purchased from Sigma (MO, USA); anti-CYP1B1 (A1377) and anti-FBXO10 (A14871) were obtained from Abclonal (CHN); anti-ACSL4 (22401-1-AP) was purchased from Proteintech (IL, USA); 4-HNE (MAB3249) was obtained from R&D systems (MN, USA); 12-HETE (GC40429), 20-HETE (GC35075), Arachidonic acid (GC31725), and sotrastaurin (AEB071) was obtained from GLPBIO (CA, USA).

### Cell culture and transfection

RKO, HCT116, HT29, and MC38 cell lines were obtained from American Type Culture Collection (ATCC) and were cultured in DMEM with 10% fetal bovine serum (FBS) and 1% penicillin-streptomycin in a 37 °C incubator with a humidified atmosphere containing 5% CO_2_. Cells were regularly tested for mycoplasma contamination. Transient transfection of cells was performed using Lipo3000 (Invitrogen, L3000-015) according to the manufacturer’s protocol.

### CYP1B1 knockdown cell generation

CYP1B1 knockdown cells were established by lentiviral infection. The human CYP1B1 shRNA sequence used for knockdown is as follows: sh CYP1B1-1: 5′-GCATGATGCGCAACTTCTTCA-3′; sh CYP1B1-2: 5′-GCAACTTCAGCAACTTCATCC-3′. The Mouse CYP1B1 shRNA sequence is as follows: 5′-GCAACTTCGTTCTGGACAAGT-3′.

### CCK8 assay

Cell viability was analyzed using the Cell Counting Kit-8 (CCK-8) (Beyotime, C0039, China). Briefly, cells were seeded at 10,000 cells per well in 96-well plates. Cells were treated with RSL3 (Macklin, R873890) or erastin (TOPSCIENCE, T1765) 24 h after seeding at the indicated concentrations and incubated for 24 h. Cell viability was measured according to the manufacturer’s instructions.

### C11-BODIPY assay

Cells were seeded in six-well plates and grown until 50–70% confluence in 24 h, then treated cells with 100 nm RSL3 for 6 h. Later, cells were incubated with C11-BODIPY (2.5 μM) (ThermoFisher, D3861) in fresh medium and incubated in the dark 1 h at 37 °C. Cells were then harvested while protected from light, suspended in PBS, and analyzed using flow cytometer. The gating strategies for lipid peroxidation analysis was shown in Fig. S1.

### Western blot and ubiquitination assay

For western blot, cells were harvested and lysed using RIPA buffer. Acquired proteins were separated using 10% SDS-PAGE gel electrophoresis, transferred onto a PVDF membrane and blocked with 5% skim milk. Thereafter, proteins were detected using the indicated antibodies.

For the ubiquitination assay, cells were transfected with the indicated plasmid for 48 h and treated with 20 μM MG132 for 6 h. Thereafter, cells were harvested with EBC buffer and sonication was used to disrupt the cell suspension. Cell suspensions were centrifuged at 12,000 × *g* for 15 min, the supernatant was collected and incubated with Flag antibody-conjugated sepharose beads, while rotating at 4 °C for at least 12 h. Samples were washed thrice with EBC buffer, beads were added to 2× loading buffer and boiled for 6 min, then subjected to western blot analysis with indicated antibody.

### Quantitative real-time polymerase chain reaction (qPCR)

Total RNA was isolated from the indicated cell lines using Total RNA Extraction Kit (Solarbio, R1200) according to the manufacturer’s instructions. The RNA sample was reverse transcribed into cDNA using the PrimeScript^TM^ RT Reagent Kit (RR047A, TaKaRa, Tokyo, Japan) and specific primers were used to amplify DNA. Briefly, mRNA levels were quantified by qPCR, each mRNA was normalized to GAPDH as the endogenous control for all experiments. The primer sequences used in the qPCR assays are as follows: CYP1B1: 5′-TGAGTGCCGTGTGTTTCGG-3′ and 5′-GTTGCTGAAGTTGCGGTTGAG-3′; ACSL4: 5′-CATCCCTGGAGCAGATACTCT-3′ and 5′-TCACTTAGGATTTCCCTGGTCC-3′; GAPDH: 5′-GGAGCGAGATCCCTCCAAAAT-3′ and 5′-GGCTGTTGTCATACTTCTCATGG-3′.

### Animals and tumor models

Female C57BL/6J mice (approximately aged 6 weeks) were purchased from Guangdong Medical Laboratory Animal Center (Guangzhou, China) and maintained under specific pathogen-free conditions. Mice were randomly divided into four groups without any selective criteria, and MC38 cell (1 × 10^6^) with stably expressing ctrl shRNA or CYP1B1 shRNA were implanted into the right flank of mice and allowed to grow six days before treatment, each group has five mice (*n* = 5). Mouse anti-PD-1 antibody (100 μg; BE0146, Bio X Cell) or isotype control IgG antibody (BE0083, Bio X Cell) was injected intraperitoneally, twice a week, for five injections in total. Tumor volume was measured according to the following formula: Volume = (Length × Width^2^)/2. The animal experiment was approved by the Institutional Animal Care and Use Committee of Jinan University. The investigator was not blinded in animal study.

### Immunohistochemical (IHC) staining

A total of 44 human CRC tissues were collected and embedded in paraffin. IHC staining was performed according to the R.T.U. Vectastain Kit (Vector Laboratories) instruction. IHC staining was evaluated according to immunescore based on the percentage of positive cells and staining intensity. The staining intensity was defined as 0–4 (4 being the strongest). The immunoscore was calculated by multiplying the intensity by positive cell percentage, producing a total range of 0–400. The human CRC tissues in this study were approved by the Clinical Ethics Committee of the First Affiliated Hospital of Jinan University, and written informed consent was obtained from the patients before inclusion in the study.

### Statistical analysis

Data were obtained from at least three independent experiments. All statistical analyses were performed using GraphPad Prism 8.3.0. Unless otherwise mentioned, all data were presented as mean ± SD. Student’s two-sided *t* test was performed to compare the statistical significance. All *p* values were two-tailed and *p* values < 0.05 were considered statistically significant.

## Results

### CYP1B1 renders CRC cells resistant to ferroptosis

**Fig. 1:**
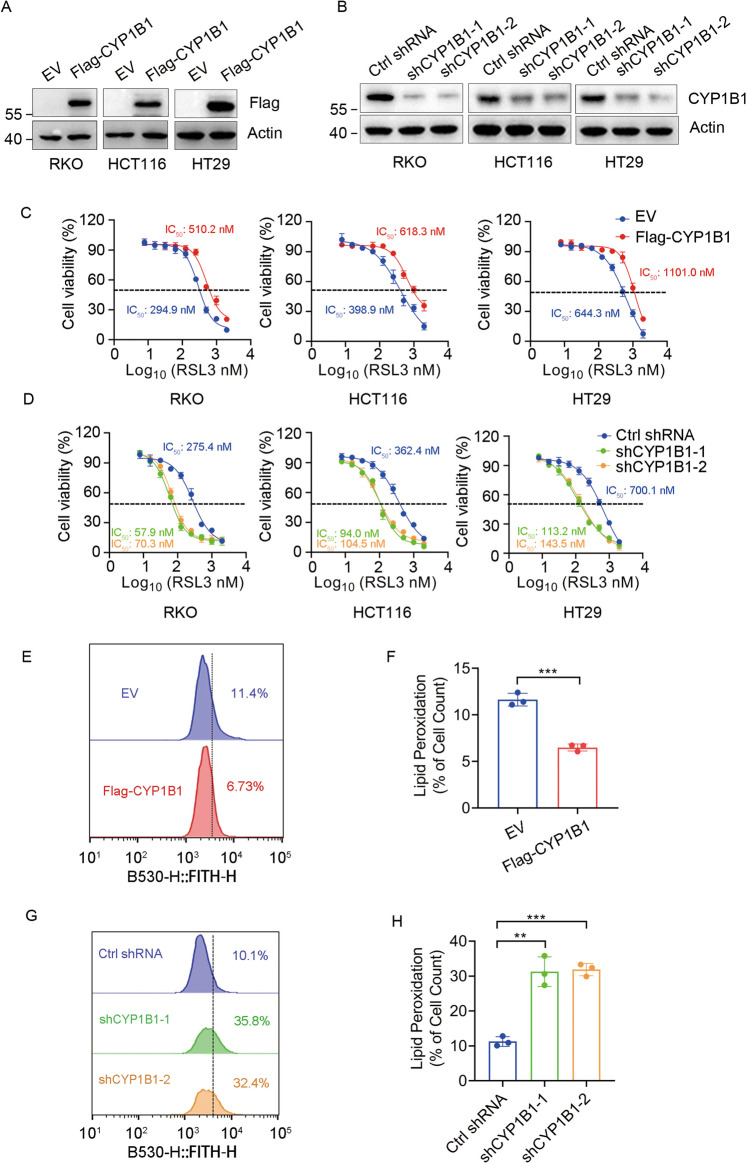
CYP1B1 is involved in ferroptosis. **A** Western blot analysis of RKO, HCT116 and HT29 cells expressing empty vector (EV) or Flag-CYP1B1. **B** Western blot analysis of RKO, HCT116, and HT29 cells expressing control (ctrl) shRNA or CYP1B1 shRNA. **C**, **D** Viability curves of RKO, HCT116 and HT29 cells with CYP1B1 overexpression or knockdown treated with the indicated concentrations of RSL3. **E**–**H** Lipid peroxidation levels were tested using C11-BODIPY in CYP1B1 overexpression (**E**, **F**) or knockdown (**G**, **H**) RKO cells. The data shown represents the mean ± SD; *n* = 3. *p* value was determined by Student’s two-sided *t* test. **p < 0.01, ***p < 0.001.

To test whether CYP1B1 is involved in ferroptosis, we generated CYP1B1 overexpressed or knockdown cells in RKO, HCT116, and HT29 cell lines (Fig. 1A, B), and tested the drug sensitivity of these cells with RSL3, an established ferroptosis inducer. As expected, cells with ectopic expression of CYP1B1 were resistant to RSL3 (Fig. 1C). Conversely, CYP1B1 knockdown led to cells being more sensitive to RSL3 (Fig. 1D). It was consistent that these effects were also found when cell were treated with erastin (Fig. S2). Meanwhile, we measured membrane lipid hydroperoxide levels in RKO cells using BODIPY-C11 oxidation. RKO cells overexpressing CYP1B1 exhibited reduced lipid peroxidation (Fig. 1E, F). In contrast, membrane lipid hydroperoxide levels in CYP1B1 knockdown cells was markedly raised (Fig. 1G, H). Together, these results suggested that CYP1B1 expression alleviated lipid peroxidation to protect cells from ferroptosis and contributed to ferroptosis resistance.

### CYP1B1 promotes ACSL4 ubiquitination and degradation

**Fig. 2:**
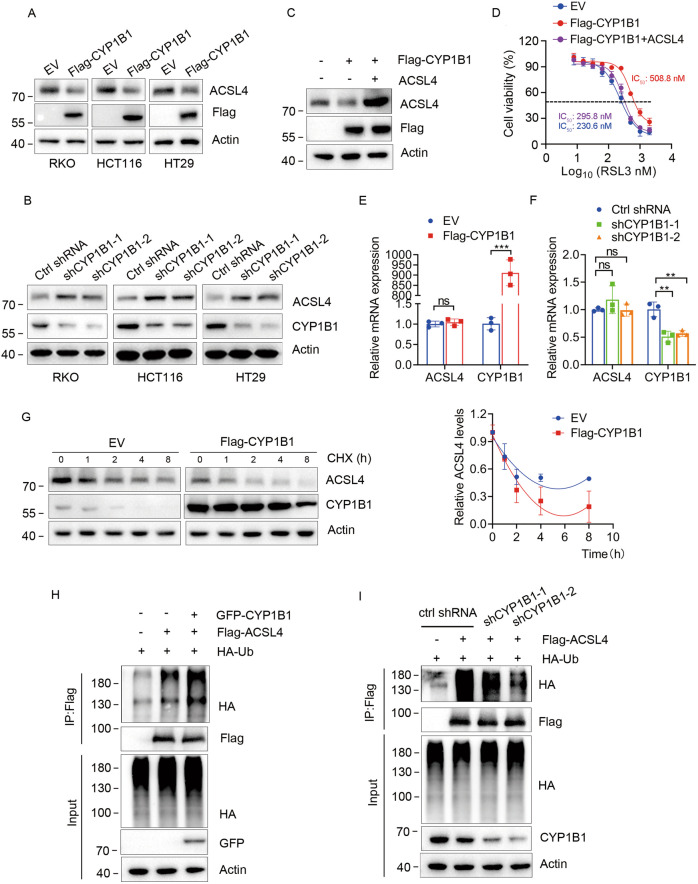
CYP1B1 regulates ferroptosis by mediating ACSL4. **A**, **B** RKO, HCT116 and HT29 cells with CYP1B1 overexpression or knockdown via lentiviral infection, followed by western blot using the indicated antibodies. **C** Western blot analysis of RKO cells were transfected with empty vector (EV) or ACSL4 plasmids. **D** Viability curves of RKO cells treated with indicated RSL3 concentrations. **E**, **F** The mRNA levels of RKO cells with CYP1B1 overexpression or knockdown were analyzed using qPCR. The data shown represents the mean ± SD. *n* = 3. *p* value was determined by student’s two-sided *t* test. **p < 0.01, ***p < 0.001. **G** RKO cells expressing EV or Flag**-**CYP1B1 were treated with 100 μg/ml CHX and harvested at the indicated times. Cells were lysed and analyzed using western blot with the indicated antibodies (left). Quantification of ACSL4 protein levels was shown (right). The data shown represent the mean ± SD. *n* = 3. **H** RKO cells were transfected with Flag-ACSL4, GFP-CYP1B1 and HA-Ub, after 48 h, treated with 5 μM MG132 for 6 h, then analyzed for ACSL4 ubiquitination. **I** CYP1B1 ctrl or knockdown RKO cells were transfected with Flag-ACSL4 and HA-Ub for 48 h and treated with 5 μM MG132 for 6 h before harvesting for ubiquitination analysis.

Next, we explored the mechanistic role of CYP1B1 in ferroptosis resistance. CYP1B1 is a member of the CYP superfamily and participates in metabolic events, including the metabolism of fatty acids [8]. We questioned whether CYP1B1 induced ferroptosis resistance by regulating the expression of acyl-CoA synthetase long-chain family member 4 (ACSL4), a critical enzyme that participates in the esterification of CoA to free fatty acids and regulates metabolic lipid reprogramming [4, 12]. Western blot analysis showed that CYP1B1 overexpression decreased ACSL4 protein levels (Fig. 2A), while CYP1B1 knockdown increased ACSL4 protein levels in RKO, HCT116, and HT29 cells (Fig. 2B). Next, we investigated whether CYP1B1 suppression of ferroptosis was the result of ACSL4 downregulation. We transfected CYP1B1 overexpressed RKO cells with empty vector (EV) or ACSL4 plasmids (Fig. 2C), and conducted the CCK8 assay. Results revealed that the exogenous expression of ACSL4 reversed cellular resistance to RSL3 (Fig. 2D), suggesting that CYP1B1 suppressed ferroptosis in an ACSL4-dependent manner.

Thereafter, we examined the mRNA changes of ACSL4. Interestingly, we failed to detect changes to ACSL4 mRNA levels in cells with CYP1B1 overexpression or knockdown (Fig. 2E, F). These results indicated that CYP1B1 regulates ACSL4 proteins at the post-translational level. To test whether CYP1B1 affects ACSL4 protein stability, EV or CYP1B1 overexpressed RKO cells were treated with cycloheximide (CHX) to inhibit protein synthesis for indicated time points. Western blot results showed that CYP1B1 overexpression decreased the half-life of ACSL4 (Fig. 2G). Based on these results, we considered that CYP1B1 decreased ACSL4 protein stability by increasing the ubiquitination level of ACSL4. Next, we analyzed the effect of CYP1B1 on the ubiquitination of ACSL4. As expected, we found that CYP1B1 overexpression increased poly-ubiquitin chain linked-ACSL4 (Fig. 2H), whereas CYP1B1 knockdown reduced the polyubiquitination level of ACSL4 (Fig. 2I). These results suggest that CYP1B1 promoted ACSL4 degradation by increasing ACSL4 ubiquitination.

### 20-HETE promotes FBXO10 expression and is critical for CYP1B1-induced ACSL4 degradation

**Fig. 3:**
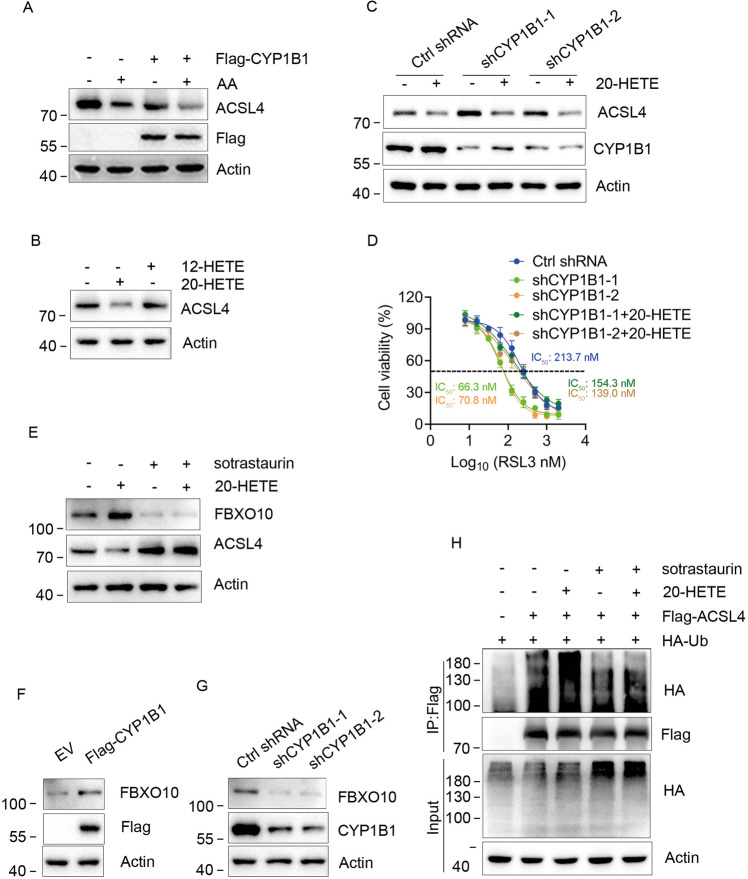
20-HETE is responsible for the ubiquitination and degradation of ACSL4. **A** RKO cells expressing EV or Flag-CYP1B1 were treated with 10 μM AA for 24 h, then analyzed by western blot. **B** RKO cells were treated with 10 μM 12-HETE or 20-HETE for 24 h and analyzed using western blot. **C** RKO cells expressing ctrl or CYP1B1 shRNAs were treated with 10 μM 20-HETE for 24 h, followed by western blot analysis. **D** Viability curves of RKO cells treated with 20-HETE and indicated concentrations of RSL3. **E** RKO cells were treated with 10 μM 20-HETE and/or sotrastaurin for 24 h, followed by western blot analysis. **F**, **G** FBXO10 expression was analyzed using western blot in RKO cells with CYP1B1 overexpression or knockdown. **H** RKO cells were transfected with Flag-ACSL4, GFP-CYP1B1 and HA-Ub and treated with 10 μM 20-HETE and/or sotrastaurin for 24 h, and 5 μM MG132 for 6 h before harvesting. Thereafter, poly-ubiquitinated ACSL4 was analyzed using western blot.

ACSL4 catalyzes AA to arachidonyl-CoA, thus promoting ferroptosis. Exogenous AA can enhance ferroptosis induced by RSL3 [12]. Meanwhile, CYP1B1 metabolizes AA to hydroxyeicosatetraenoic acids (HETEs) [13]. Therefore, we questioned whether CYP1B1 reduced ACSL4 protein level by downregulating intracellular AA. RKO cells were transfected with EV or Flag-CYP1B1 plasmids and treated with 10 μM AA for 24 h. Western blot showed that AA did not rescue ACSL4 expression in CYP1B1 overexpressed cells, and ACSL4 expression appeared reduced (Fig. 3A). According to this result, we speculated that CYP1B1 may protect cells from ferroptosis by degrading ACSL4 via metabolites of AA. The metabolites of AA produced by CYP1B1 are mainly 12-HETE and 20-HETE in humans [13], thus, we next tested whether CYP1B1-induced ACSL4 downregulation was due to 12-HETE or 20-HETE. RKO cells were treated with 10 μM 12-HETE or 20-HETE for 24 h and ACSL4 protein levels were determined using western blot. The results showed that 20-HETE, but not 12-HETE decreased ACSL4 protein levels (Fig. 3B). To identify whether the increase in ACSL4 protein levels caused by CYP1B1 knockdown was due to 20-HETE, we treated CYP1B1 knockdown RKO cells with 10 μM 20-HETE for 24 h and detected the ACSL4 protein level. As shown in Fig. 3C, 20-HETE decreased ACSL4 protein levels and reversed the CYP1B1 knockdown-induced increase in ACSL4 protein. In addition, 20-HETE, but not 12-HETE reduced the sensitivity of CYP1B1 knockdown RKO cells to RSL3 (Figs. 3D and S3A). Taken together, these data suggest that CYP1B1 down regulates ACSL4 protein and promotes ferroptosis tolerance through 20-HETE.

20-HETE is known to activate the PKC signal pathway to trigger downstream signaling and the transcription of target genes [14–16]. We aimed to determine whether 20-HETE promoted the expression of F-box only protein 10 (FBXO10), a reported E3 ubiquitin ligase of ACSL4 [17]. We found that 20-HETE increased the expression of FBXO10, and this increase was eliminated by sotrastaurin (a PKC inhibitor), while ACSL4 showed the opposite trend (Fig. 3E). In addition, exogenous expression of CYP1B1 increased FBXO10 protein level, while CYP1B1 knockdown decreased FBXO10 expression (Fig. 3F, G). As Bcl-2 was reported a FOXO10 target in lymphoma [18], we test whether CYP1B1 can affect the expression of Bcl-2 in CRC cells. Results showed that CYP1B1 had no obvious effect on Bcl-2 expression (Fig. S3B, C). It may be that FBXO10 plays different roles in different cells. Next, we examined whether the polyubquitination level of ACSL4 was also regulated by 20-HETE and the PKC inhibitor. As shown in Fig. 3H, 20-HETE increased the poly-ubiquitin chain linked-ACSL4, while the polyubquitination level of ACSL4 decreased in cells treated with sotrastaurin only or combined with 20-HETE. Collectively, these data demonstrated that 20-HETE, a metabolite of AA, enhances ACSL4 degradation by activating the PKC signaling pathway and inducing the expression of E3 ubiquitin ligase FBXO10.

### CYP1B1 expression attenuates the efficacy of anti-PD-1 therapy

**Fig. 4:**
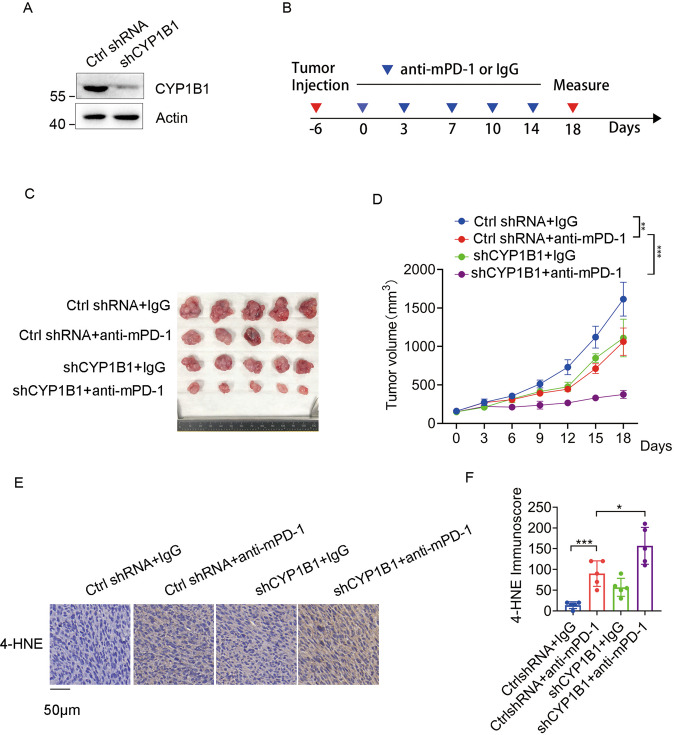
CYP1B1 expression attenuates the effect of anti-PD-1 therapy. **A** Western blot analysis of MC38 cells expressing ctrl shRNA or CYP1B1 shRNAs. **B** C57BL/6 J mice carrying the indicated MC38 cell formed tumors at the right flank were treated with anti-mPD-1 antibody or isotype control IgG for a total of five treatments. **C**, **D** Tumor images **C** and tumor growth curve **D** in mice bearing indicated MC38 cell tumors treated with anti-mPD-1 or isotype control IgG antibodies. **E** IHC staining of 4-HNE in MC38 tumors expressing ctrl or CYP1B1 shRNA. **F** Quantification of 4-HNE IHC staining. *n* = 5 per group. *p* value was determined by Student’s two-sided *t* test. *p < 0.05, **p < 0.01, ***p < 0.001.

Ferroptosis plays an important role in cancer immunotherapy [3, 4], thus we aimed to determine whether CYP1B1 disrupts the effectiveness of anti-PD-1 therapy. CYP1B1 was knocked down in MC38 cells using lentiviral infection and western blot was used to analyze protein expression (Fig. 4A). We subcutaneously injected MC38 cells with stably expressing ctrl shRNA or CYP1B1 shRNA into the right flank of C57BL/6 J mice, and treated these mice with anti-mPD-1 antibody or isotype control IgG according to the treatment schedule (Fig. 4B). Anti-mPD-1 treatment significantly inhibited tumor growth in mice bearing CYP1B1 knockdown tumors (Fig. 4C, D). Moreover, IHC showed increased in 4-HNE expression, a lipid peroxidation marker, when mice with CYP1B1 knockdown were treated with anti-mPD-1 (Fig. 4E, F). These data suggest that CYP1B1 deficiency enhanced ferroptosis and sensitized tumor cells to anti-PD-1 therapy.

### CYP1B1 levels are negatively correlated with ACSL4 levels in CRC tissues

**Fig. 5:**
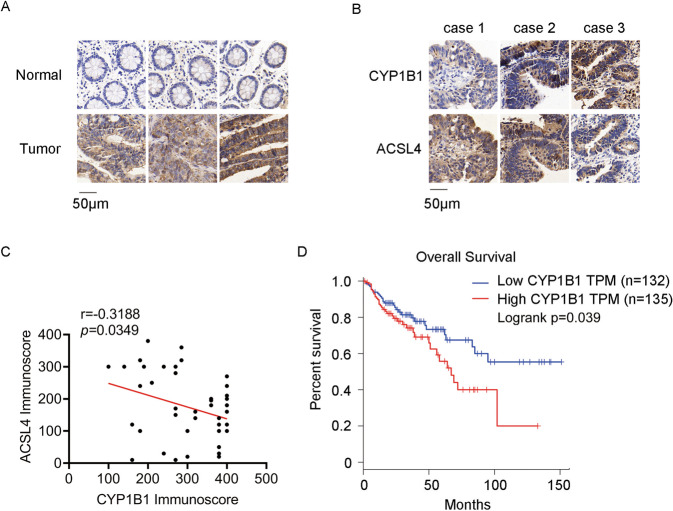
The correlation between CYP1B1 and ACSL4. **A** IHC staining of CYP1B1 in CRC cancer tissues and adjacent normal tissues. **B** IHC staining of CYP1B1 and ACSL4 in CRC cancer tissues. **C** The correlation analysis between CYP1B1 and ACSL4 in CRC cancer tissues was explored using Pearson correlation analysis (*n* = 44). **D** Overall survival was analyzed in COAD patients from the GEPIA database with low or high expression of CYP1B1 (defined by RNA sequencing with group cut-off values of 50 and 50%).

To evaluate the clinical relevance of CYP1B1 and ACSL4 in CRC tumors, we first compared the expression of CYP1B1 and ACSL4 in CRC cancer tissues and adjacent normal tissues. IHC staining showed that CYP1B1 was highly expressed in CRC cancer tissue (Fig. 5A), which is consistent with previous studies [19, 20]. Next, we determined the correlation between CYP1B1 and ACSL4 in CRC tumor tissues, estimating by semiquantitative evaluation. IHC analysis of serial sections revealed that ACSL4 protein levels were negatively correlated to CYP1B1 protein levels (Fig. 5B, C). Furthermore, we analyzed the prognostic value of CYP1B1 using the GEPIA database (http://gepia.cancer-pku.cn/index.html), and we found that high expression of CYP1B1 levels were significantly associated with poor overall survival for colon adenocarcinoma (COAD) patients (Fig. 5D). These results suggest that CYP1B1 may be useful as a potential biomarker and may provide a new therapeutic target in CRC patients.

## Discussion

**Fig. 6:**
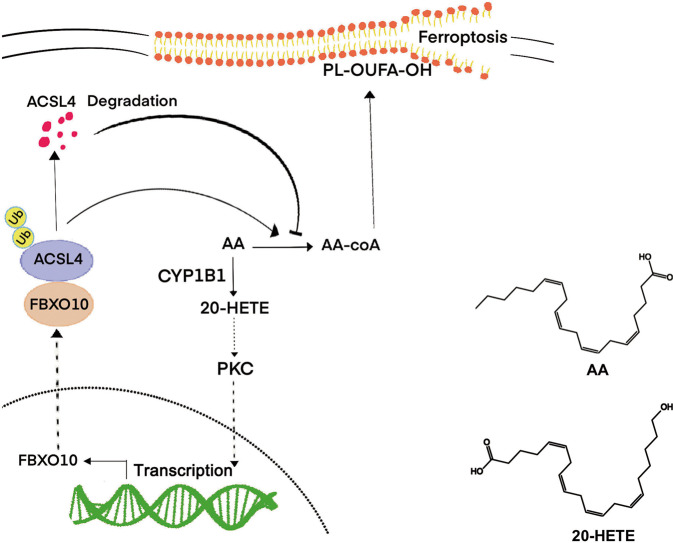
Schematic diagram of CYP1B1 inducing ACSL4 degradation and inhibiting ferroptosis. Briefly, CYP1B1 metabolizes AA to 20-HETE to activate the PKC pathway, thereby increasing FBXO10 expression, which promotes ACSL4 degradation and leads to tumor cell resistance to ferroptosis. The lower right side shows the chemical structures of AA and 20-HETE.

Ferroptosis is a type of programmed cell death characterized by lipid peroxidation, and is distinct from apoptosis, necroptosis, and autophagy. Ferroptosis relates to anticancer drug resistance, and immune surveillance, and has received extensive interest in the field of cancer therapy [6, 21, 22]. Here, we proved that CYP1B1 is an important regulator of ferroptosis and affects the sensitivity of anti-PD-1 therapy. Mechanistically, CYP1B1 induced tumor cell resistance to ferroptosis by increasing ACSL4 ubiquitination and promoting its degradation (Fig. 6).

ACSL4 is an essential determinant of ferroptosis that functions by catalyzing lipid biosynthesis for the execution of ferroptosis [12]. ACSL4 prefers AA as a substrate and catalyzes AA to arachidonyl-CoA. Phospholipids in AA may serve as primary lipid peroxidation substrates for ferroptosis. However, it seems paradoxical that AA can enhance ferroptosis, but reduces ACSL4 protein level. Interestingly, ACSL4 knockout cells are resistant to ferrotosis [4]. In this study, we reported that CYP1B1 metabolizes AA to 20-HETE, which promotes ACSL4 protein degradation by activating the PKC signaling pathway. This mechanism may explain how CYP1B1 protects tumor cells from ferroptosis. Recently, researchers have reported that CYP1B1 plays a critical role in anti-cancer drug resistance [23–26]. Additionally, the role of CYP1B1 is also described in tumor proliferation and metastasis [27, 28].

Zou and colleagues reported that cytochrome P450 oxidoreductase (POR) promoted ferroptosis by donating electrons to cytochrome P450 (CYP) isoenzyme, while CYPs catalyzed the peroxidation of polyunsaturated fatty acids [29–32]. However, they considered the functional redundancy of CYP members, because they failed to identify the specific CYP isozymes that participated in ferroptosis via CRISPR/Cas9. In our study, we demonstrated that CYP1B1 catalyzes the conversion of AA to 20-HETE, and 20-HETE acts as a signaling molecule activating the known PKC pathway to promote FBXO10 expression and degrade ACSL4. Moreover, 20-HETE reportedly activates multiple protein kinases, promoting tumor cell survive and inflammation via signaling cascades [14, 33–35].

In conclusion, our study demonstrates that CYP1B1 promotes CRC cell resistance to ferroptosis, and CYP1B1 derived 20-HETE is responsible for CYP1B1-mediated ferroptosis resistance. In addition, CYP1B1 protein level was negatively correlated to ACSL4 level in CRC. Patients with high expression of CYP1B1 displayed poor prognosis. CYP1B1 is the first CYP superfamily member identified to regulate ferroptosis and resistance to anti-PD-1 therapy. Up to date, the inhibitors of CYP1B1 have not yet been used clinically. Further experiments are needed to clarify the role of CYP1B1 and its specific inhibitors in vitro and in vivo. Overall, our results are significant to clinical work and indicate that CYP1B1 may serve as a promising therapeutic target to enhance anti-PD-1 therapy in CRC.

## Supplementary information

Figure S1

Figure S2

Figure S3

Supplementary Figure Legends

aj-checklist

original western blots

## Data Availability

All data generated or analyzed during this study are included in this published article and its additional files.
