# Supplementary figures and images for: CYP1B1 inhibits ferroptosis and induces anti-PD-1 resistance by degrading ACSL4 in colorectal cancer
Source: Cell Death Dis. 2023 Apr 14;14(4):271. doi: 10.1038/s41419-023-05803-2 (PMC10104818; doi:10.1038/s41419-023-05803-2)

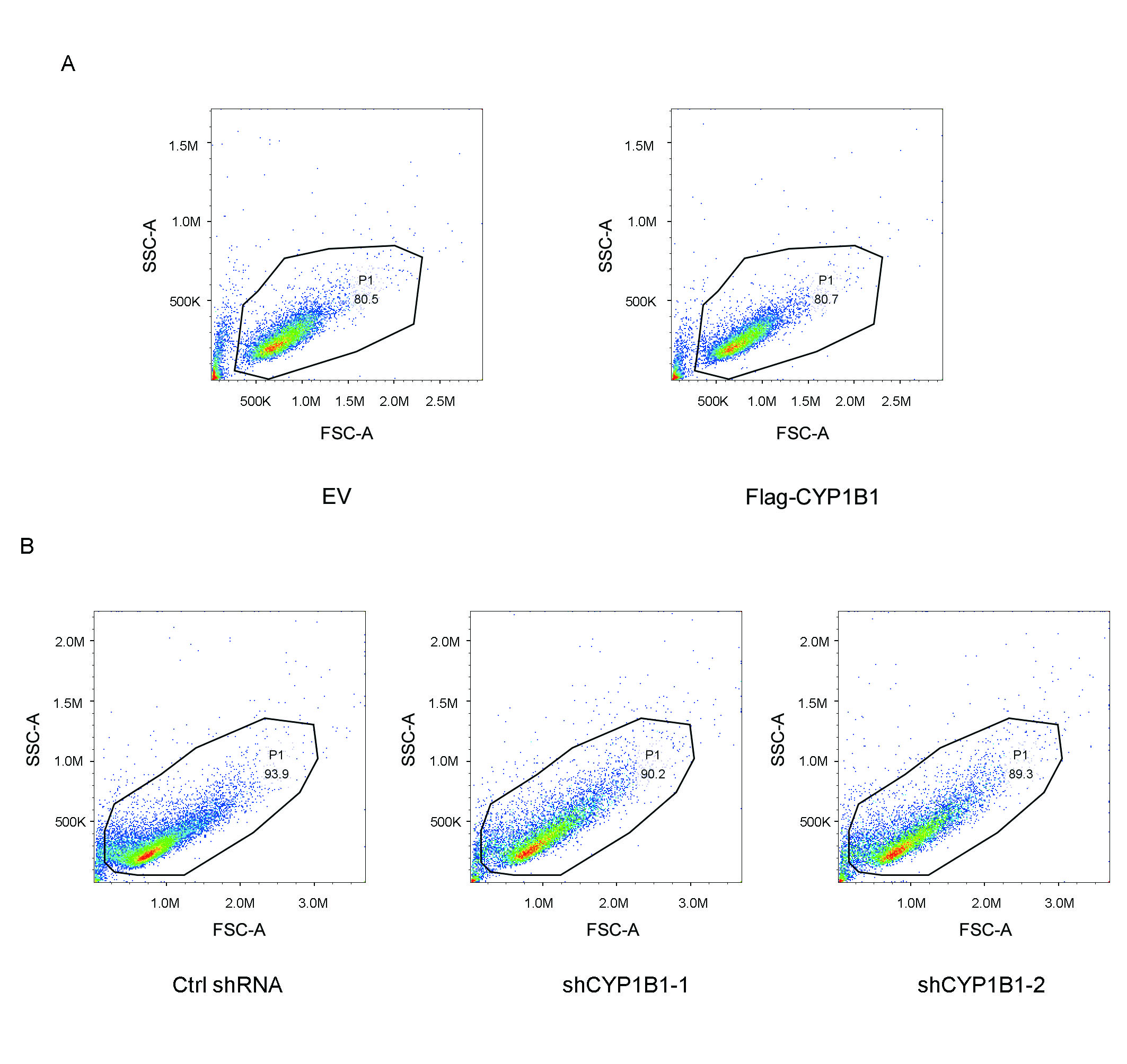

Supplement: Supplementary file 1 — Figure S1 [file 41419_2023_5803_MOESM1_ESM.jpg]

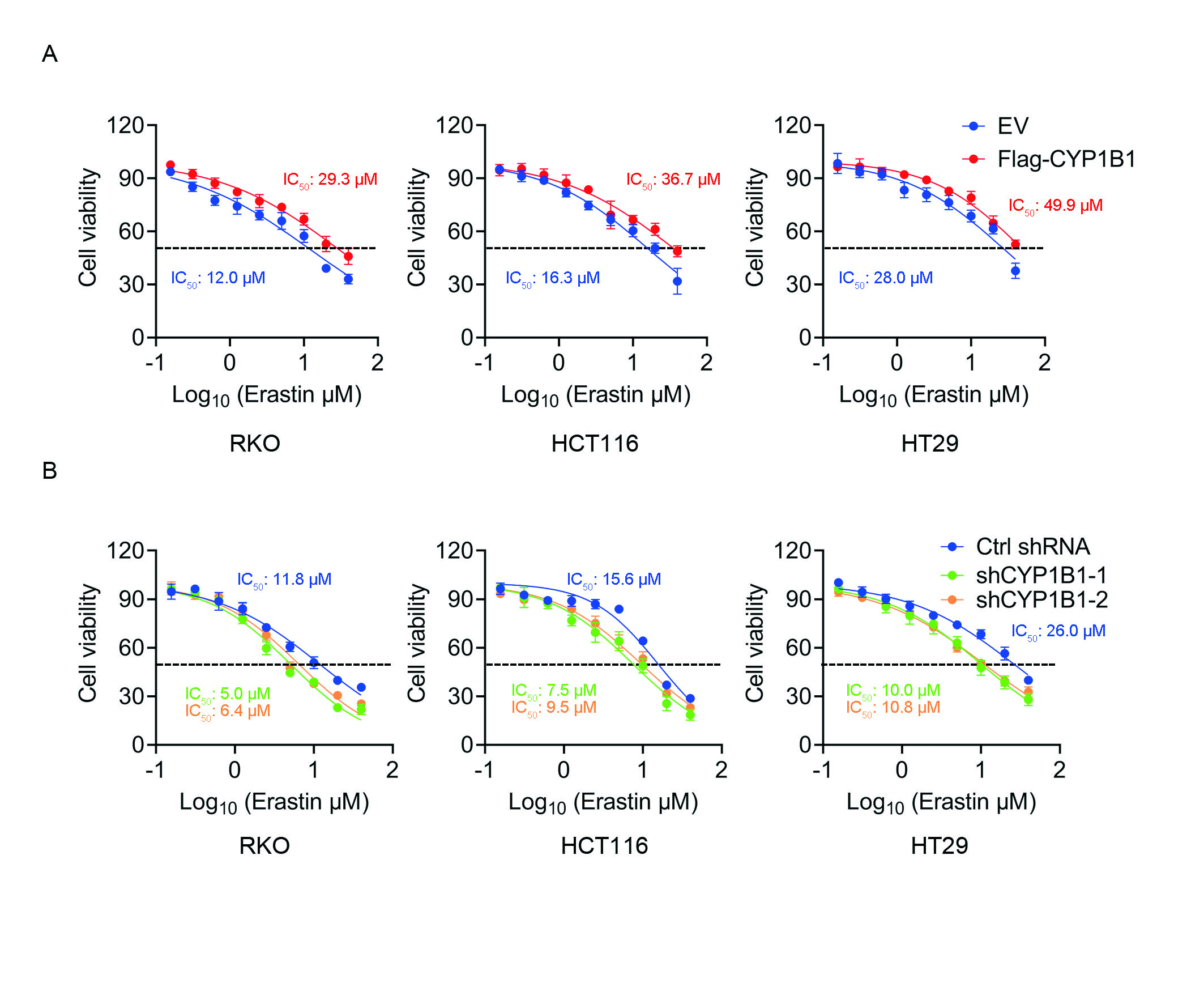

Supplement: Supplementary file 2 — Figure S2 [file 41419_2023_5803_MOESM2_ESM.jpg]

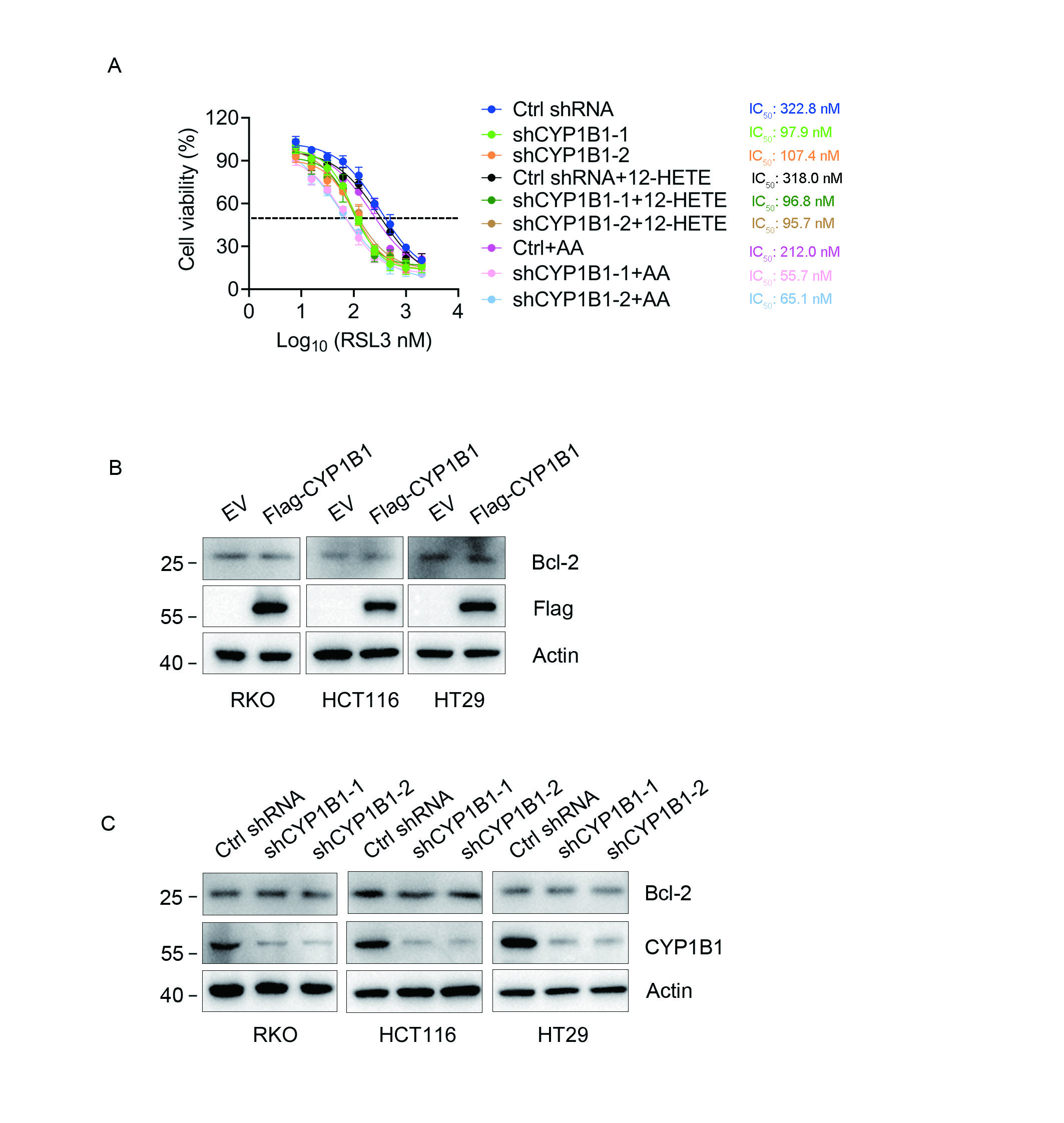

Supplement: Supplementary file 3 — Figure S3 [file 41419_2023_5803_MOESM3_ESM.jpg]
