# Supplementary material for: CYP1B1 inhibits ferroptosis and induces anti-PD-1 resistance by degrading ACSL4 in colorectal cancer
Source: Cell Death Dis. 2023 Apr 14;14(4):271. doi: 10.1038/s41419-023-05803-2 (PMC10104818; doi:10.1038/s41419-023-05803-2)
Supplement: Supplementary file 4 — Supplementary Figure Legends [file 41419_2023_5803_MOESM4_ESM.doc]

Supplementary Figure Legends

**Fig. S1 A** Gating strategies of RKO cells expressing EV or Flag-CYP1B1. **B** Gating strategies of RKO cells with expressing ctrl shRNA or CYP1B1 shRNAs.

**Fig. S2 A, B** Viability curves of RKO, HCT116 and HT29 cells with CYP1B1 overexpression **(A)** or knockdown **(B)** treated with the indicated concentrations of erastin.

**Fig. S3** **A** Viability curves of RKO cells treated with indicated concentrations of RSL3 and AA or 12-HETE. **B, C** The effect of CYP1B1 overexpression **(B)** or knockdown **(C)** on FBXO10 expression.
