# Supplementary material for: CYP1B1 inhibits ferroptosis and induces anti-PD-1 resistance by degrading ACSL4 in colorectal cancer
Source: Cell Death Dis. 2023 Apr 14;14(4):271. doi: 10.1038/s41419-023-05803-2 (PMC10104818; doi:10.1038/s41419-023-05803-2)

Figure 1A

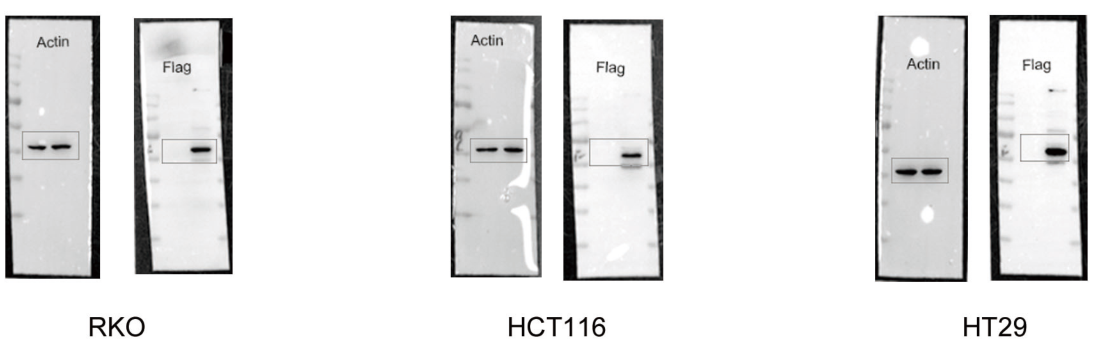

Figure 1B

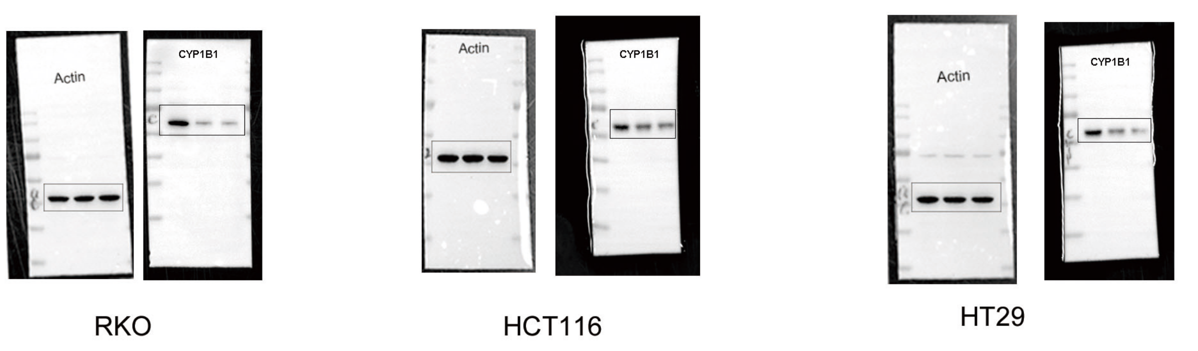

Figure 2A

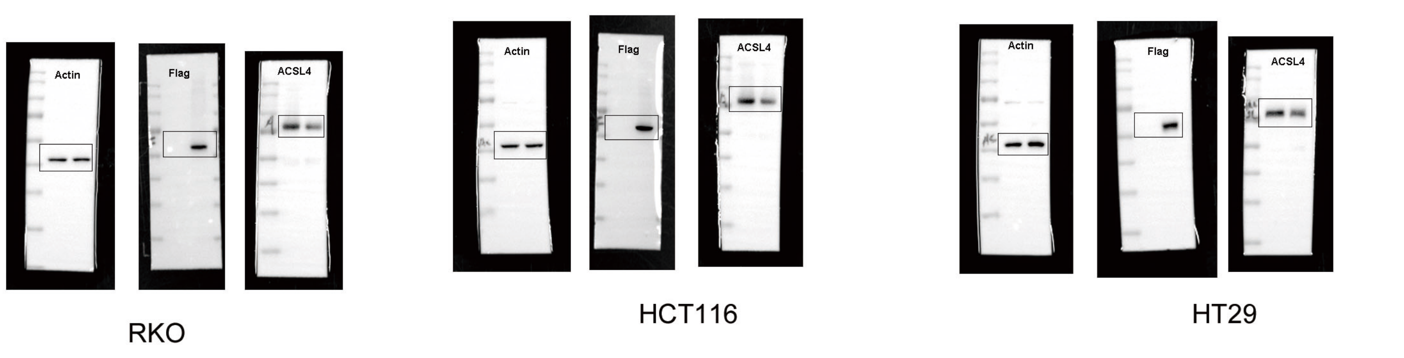

Figure 2B

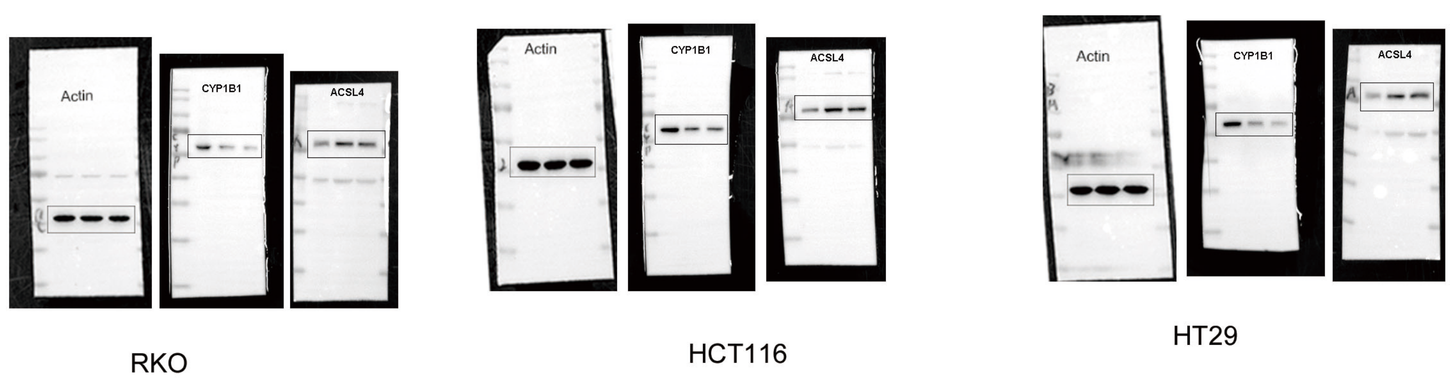

Figure 2C

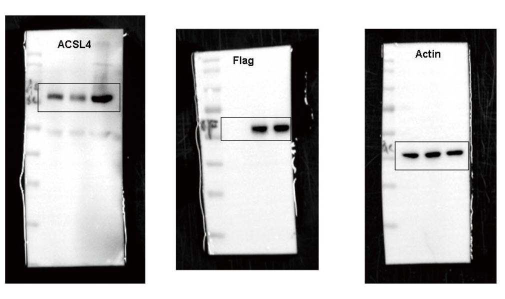

Figure 2G

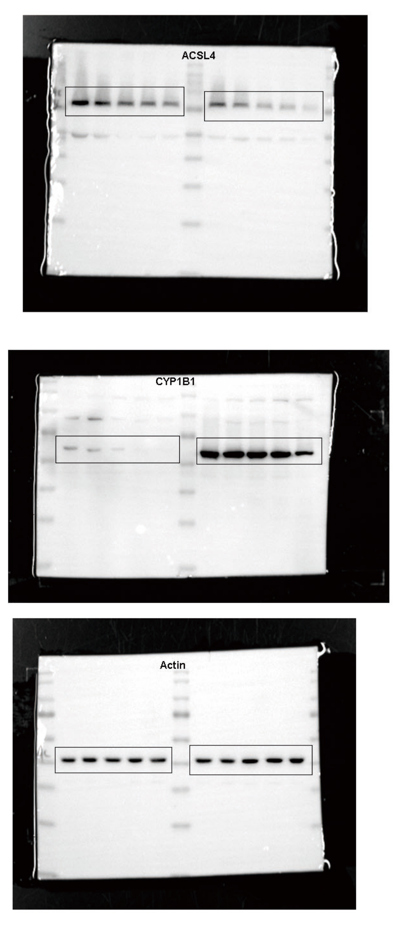

Figure 2H

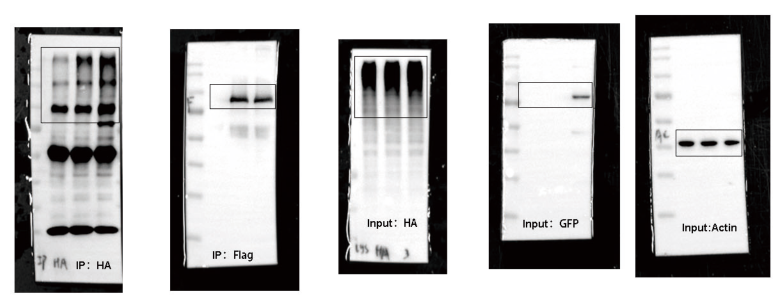

Figure 2I

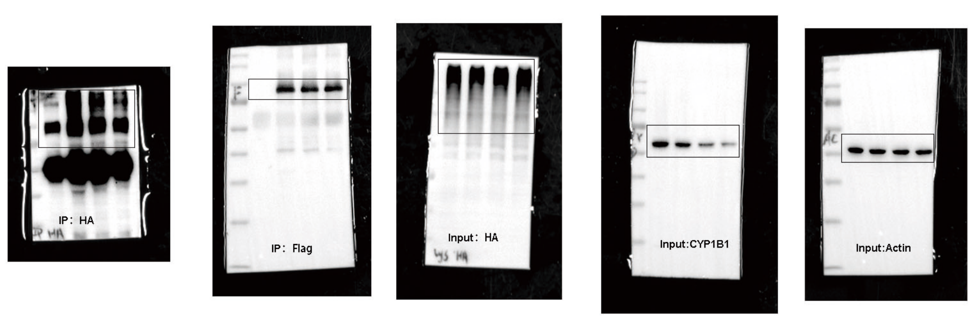

Figure 3A

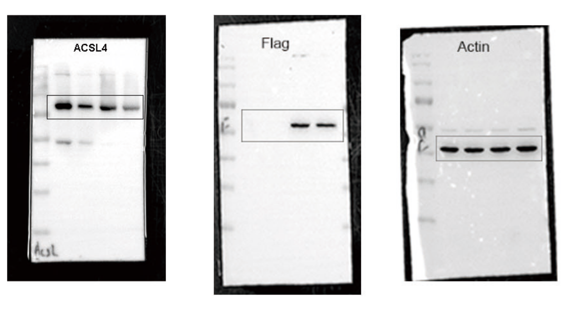

Figure 3B

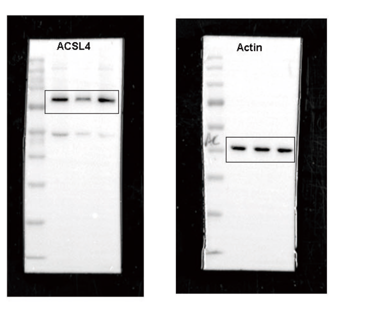

Figure 3C

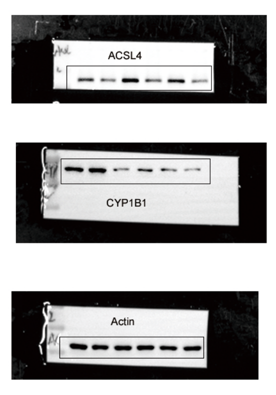

Figure 3E

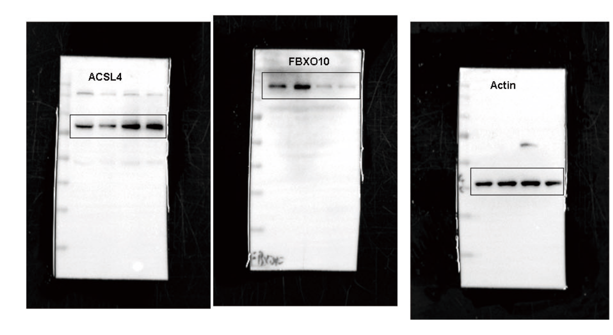

Figure 3F

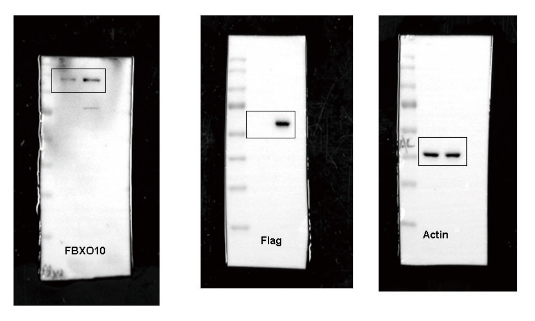

Figure 3G

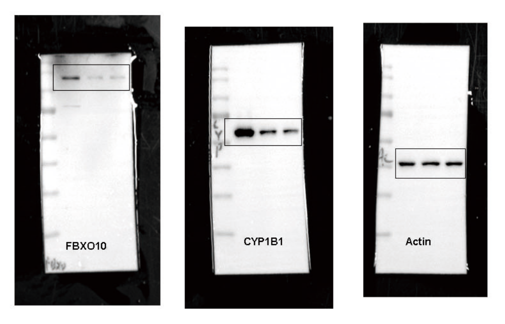

Figure 3H

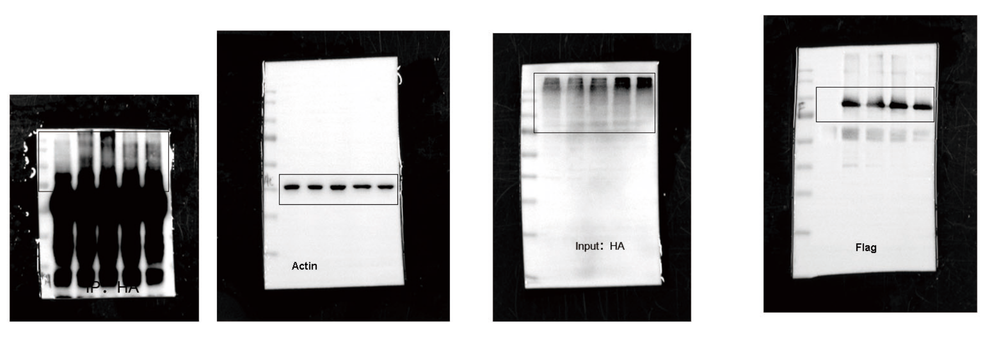

Figure 4B

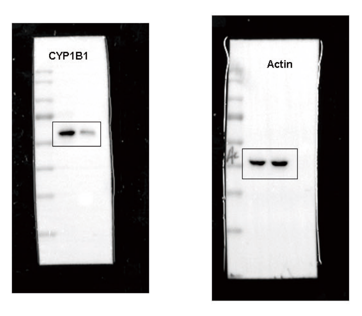

Fig S3A

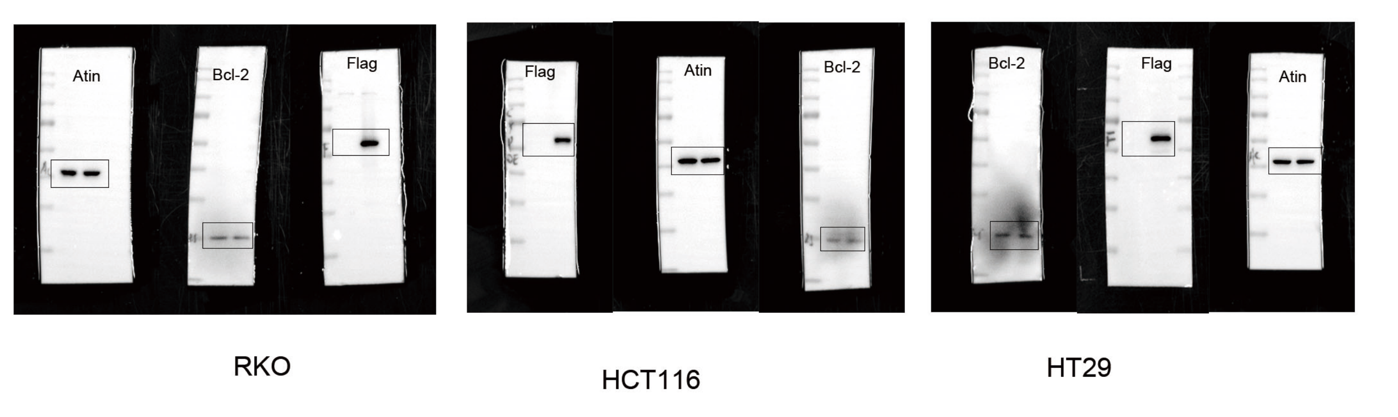

Fig S3B

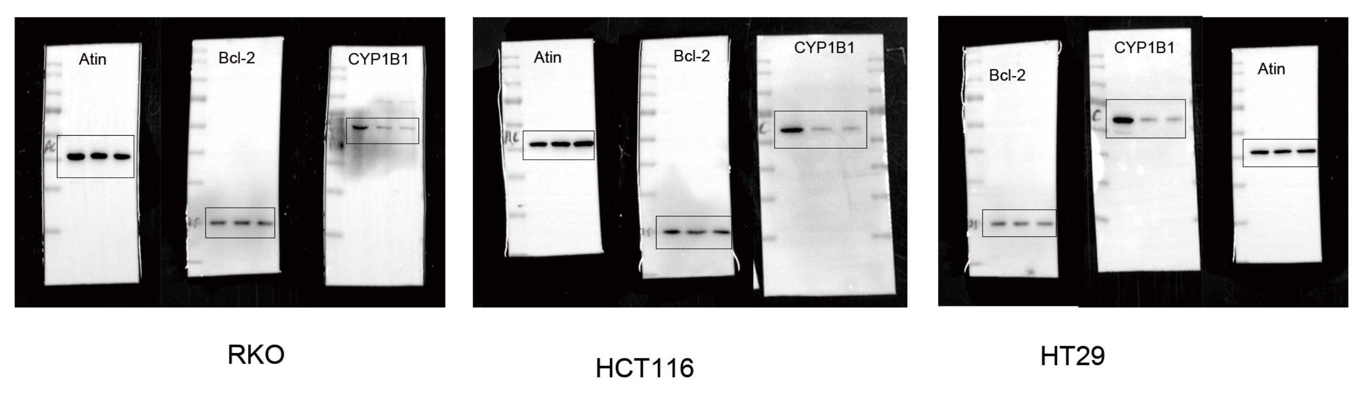

Supplement: Supplementary file 6 — original western blots [file 41419_2023_5803_MOESM6_ESM.pdf]
